# Supplementary material for: Proteome-wide in silico screening for human protein-protein interactions
Source: bioRxiv. 2025 Nov 12:2025.11.10.687652. Preprint. [Version 1] doi: 10.1101/2025.11.10.687652 (PMC12642653; doi:10.1101/2025.11.10.687652)
Supplement: 10 [file NIHPP2025.11.10.687652v1-supplement-10.pdf]

bioRxiv preprint doi: https://doi.org/10.1101/2025.11.10.687652; this version posted November 12, 2025. The copyright holder for this preprint (which was not certified by peer review) is the author/funder, who has granted bioRxiv a license to display the preprint in perpetuity. It is made available under aCC-BY 4.0 International license.

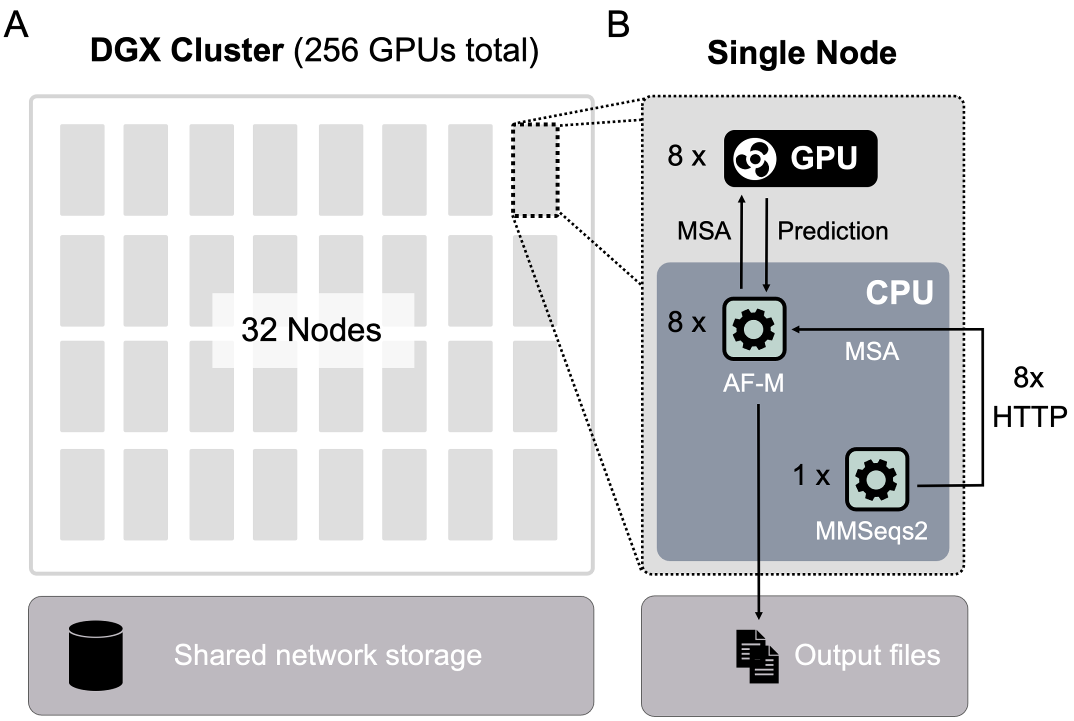

Figure S1: Computational architecture used for *in-silico* interaction screening

A. Schematic illustrating the architecture of the NVIDIA DGX cluster consisting of 32 nodes with 8 GPUs each, which was used to generate AF-M models for the 1.6 million KIRC-nominated protein pairs. **B.** A diagram displaying how various programs communicated within each node of the cluster, which operated independently and was supplied with a series of protein pairs to model. These pairs were split across the node's 8 GPUs, and multiple sequence alignments (MSAs) were supplied via HTTP requests to a node-specific instance of the MMSeqs2 server software running on the node's 8 CPUs. Once generated, predictions were deposited to a shared file system.

bioRxiv preprint doi: https://doi.org/10.1101/2025.11.10.687652; this version posted November 12, 2025. The copyright holder for this preprint (which was not certified by peer review) is the author/funder, who has granted bioRxiv a license to display the preprint in perpetuity. It is made available under aCC-BY 4.0 International license.

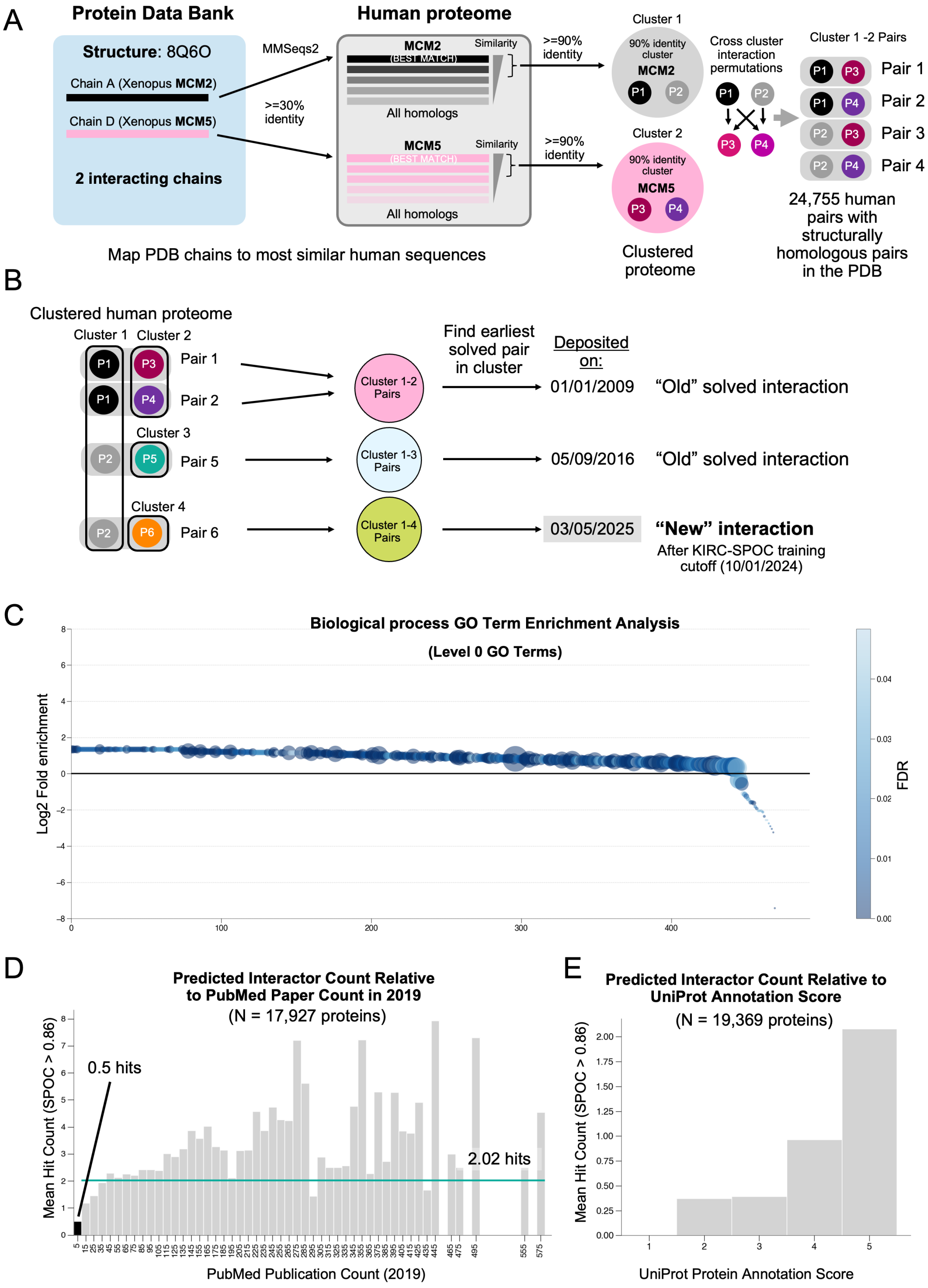

Figure S2: Analyzing the classes of proteins identified via *in-silico* screening
A. Schematic illustrating how protein interactions in the Protein Data Bank (PDB) were mapped to homologous human pairs. **B.** Schematic demonstrating how interactions were clustered by sequence and subsequently assigned a first PDB deposition date. **C.** A scatter plot of all enriched (>1) or de-enriched (<1) bioprocess GO terms (Level 0/Top level terms) associated with the 8,199 proteins in the high-confidence 16k set. The y axis is the log2 of the fold enrichment, and all terms are ranked high to low, left to right. Circle size is proportional to the number of proteins associated with that term, and circle fill color is based on the FDR associated with term enrichment analysis. **D.** Histogram of the mean number of high-confidence SPOC hits (>0.86) versus the PubMed publication count for 17,927 proteins across the screen. The horizontal teal line is the mean number of hits for all proteins with 10 or more publications in PubMed[21]. **E.** Histogram of mean number of high-confidence SPOC hits (>0.86) versus the UniProt Protein Annotation score for 19,369 proteins across the screen.

bioRxiv preprint doi: https://doi.org/10.1101/2025.11.10.687652; this version posted November 12, 2025. The copyright holder for this preprint (which was not certified by peer review) is the author/funder, who has granted bioRxiv a license to display the preprint in perpetuity. It is made available under aCC-BY 4.0 International license.

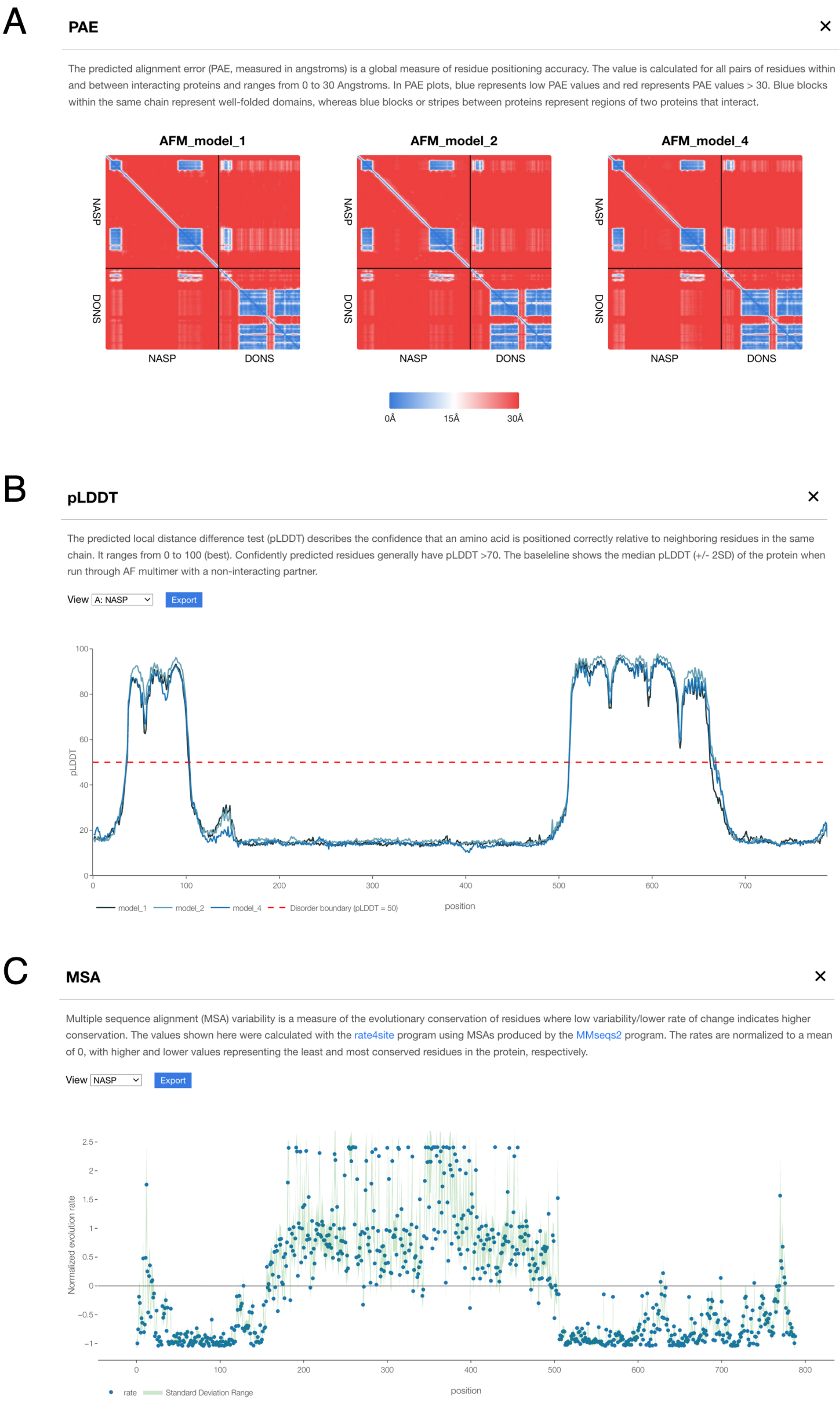

Figure S3: Additional information available at predictomes.org
Screenshot from a predicted interactor's information page at predictomes.org showing interactive **(A)** Predicted Aligned Error (PAE), **(B)** Predicted Local Distance Difference Test (pLDDT), and **(C)** Multiple Sequence Alignment (MSA) plots.

bioRxiv preprint doi: https://doi.org/10.1101/2025.11.10.687652; this version posted November 12, 2025. The copyright holder for this preprint (which was not certified by peer review) is the author/funder, who has granted bioRxiv a license to display the preprint in perpetuity. It is made available under aCC-BY 4.0 International license.

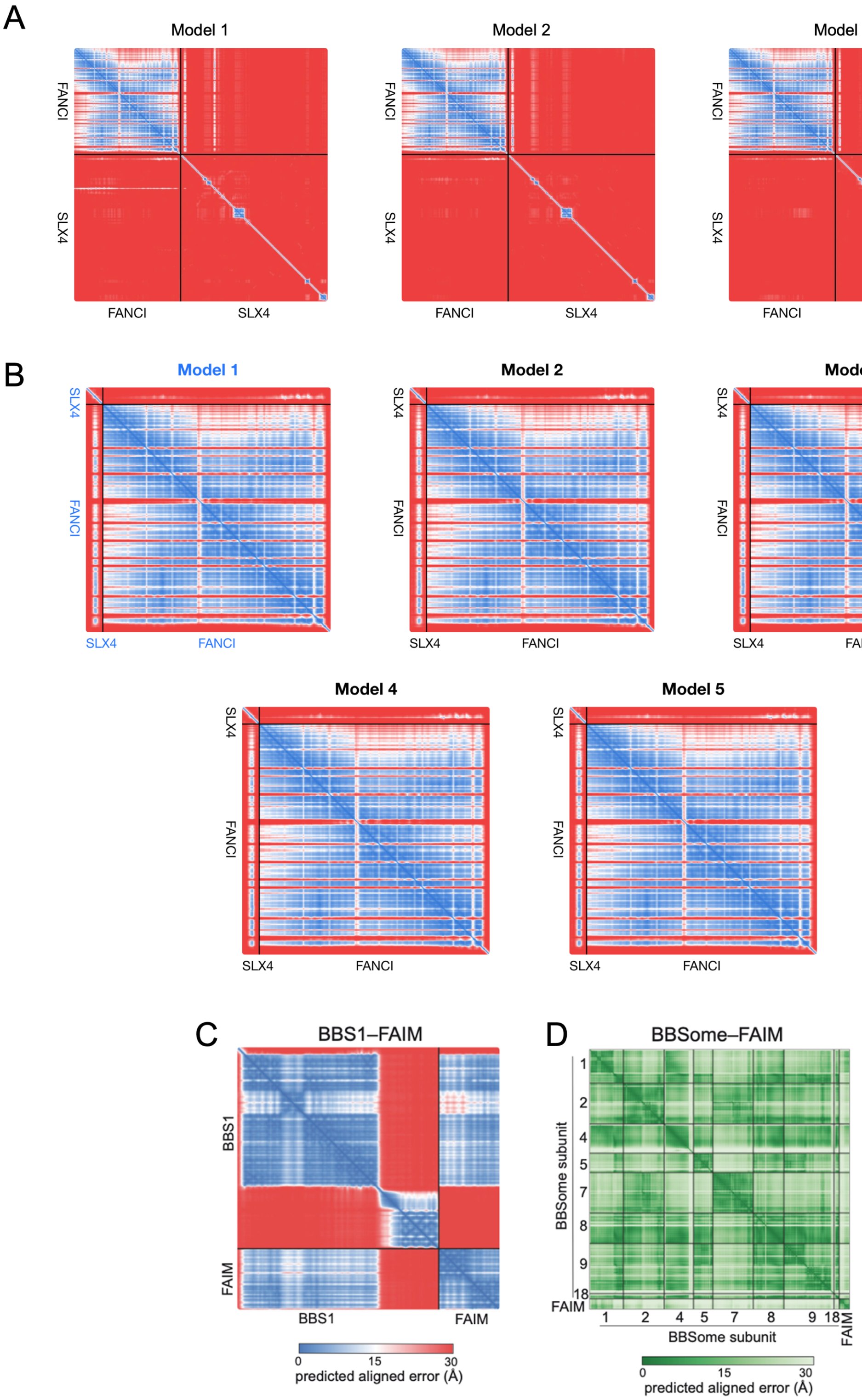

Figure S4: Predicted aligned error (PAE) plots supporting novel hypotheses
A. PAE plots depicting the FANCI–SLX4 interaction for all three predicted models retrieved from predictomes.org. The color scale indicates the per-residue predicted error, with blue representing low error and red representing high error. **B.** PAE plots showing five predicted models of the interaction between FANCI and SLX4 residues 1-100. **C.** PAE plot of a single model for the BBS1–FAIM interaction retrieved from predictomes.org. The PAE plots for the other four models look similarly confident. **D**. PAE plot of the BBSome–FAIM complex generated by AlphaFold3. In the color scale, darker green represents lower per-residue predicted error.

bioRxiv preprint doi: https://doi.org/10.1101/2025.11.10.687652; this version posted November 12, 2025. The copyright holder for this preprint (which was not certified by peer review) is the author/funder, who has granted bioRxiv a license to display the preprint in perpetuity. It is made available under aCC-BY 4.0 International license.

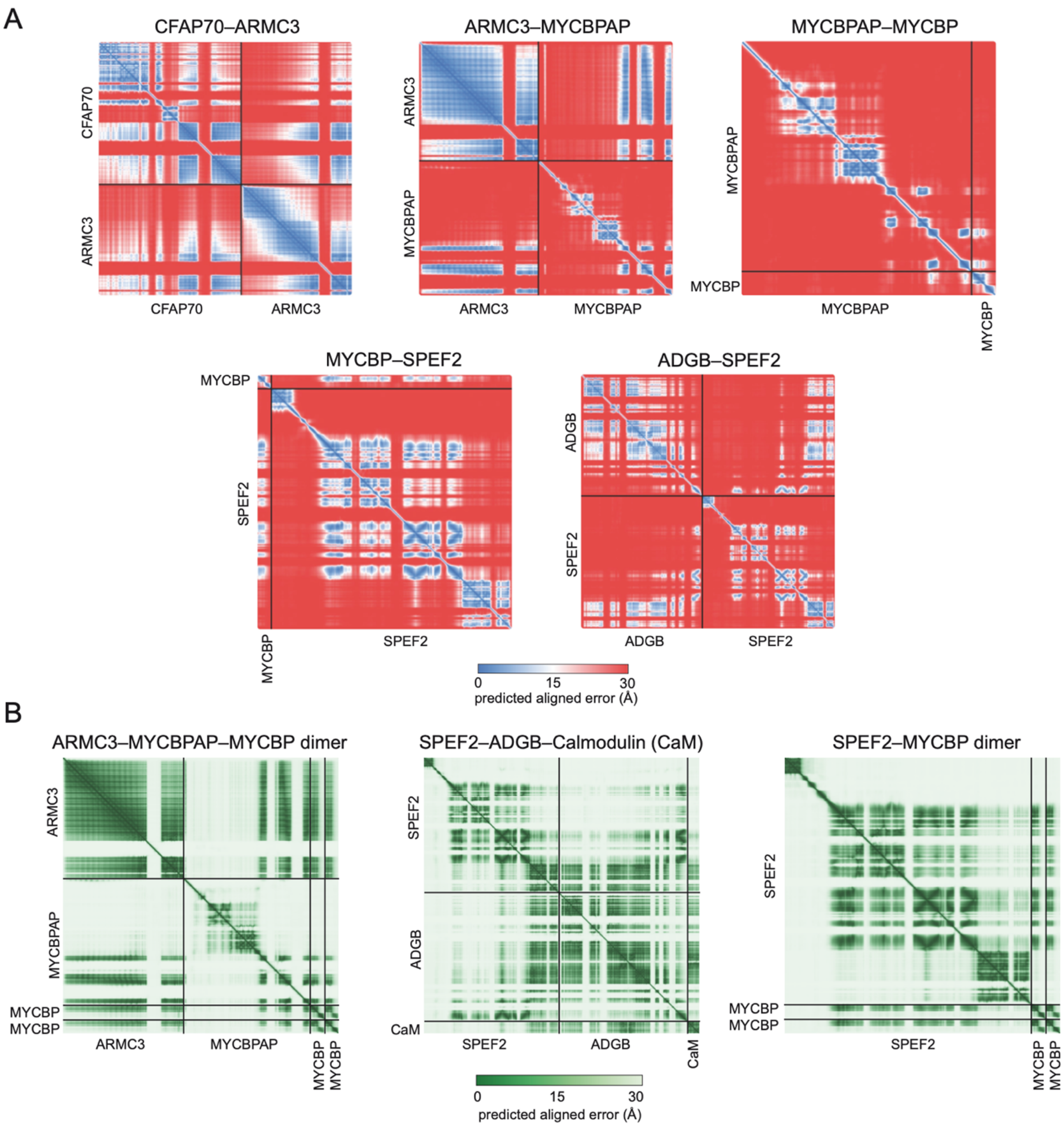

Figure S5. Predicted aligned error (PAE) plots for interactions involving central apparatus proteins.
A. PAE plots for pairwise predictions using human protein sequences retrieved from predictomes.org. Only the PAE plot for model 1 is shown for each prediction, but the other PAE plots look similarly confident. The color scale indicates the per-residue predicted error, with blue representing low error and red representing high error. **B**. PAE plots for multi-subunit predictions using AlphaFold3 with mouse protein sequences. The color scale represents the expected position error, with darker green indicating higher confidence.

bioRxiv preprint doi: https://doi.org/10.1101/2025.11.10.687652; this version posted November 12, 2025. The copyright holder for this preprint (which was not certified by peer review) is the author/funder, who has granted bioRxiv a license to display the preprint in perpetuity. It is made available under aCC-BY 4.0 International license.

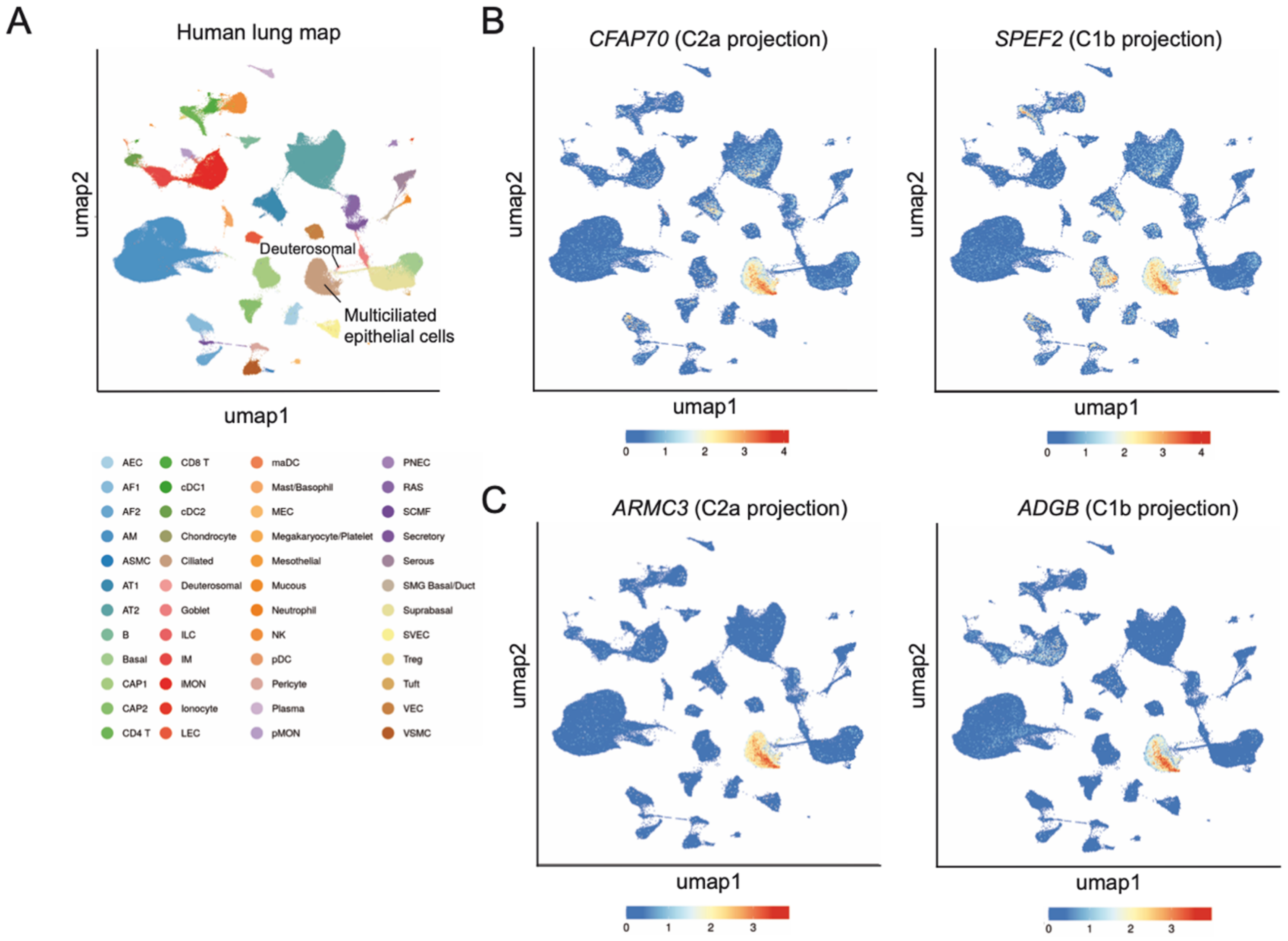

Figure S6. *ARMC3* and *ADGB* are expressed in the multiciliated cells of the lung.
A. Uniform Manifold Approximation and Projection (UMAP) plot showing clustering of major human lung cell types generated by integrating 148 single-cell or single-nucleus RNA-seq datasets from 104 human lung donors[49]. Cells are colored according to their predicted identities. Labeled are multiciliated epithelial cells and their precursor deuterosomal cells. **B**. UMAP feature plots displaying expression of *CFAP70* and *SPEF2*, with color intensity indicating normalized gene expression (blue: low, red: high). Expression is mostly restricted to the multiciliated epithelial cells, consistent with their gene products being components of the central apparatus of motile cilia. **C**. UMAP feature plots displaying the expression of *ARMC3* and *ADGB*. Both genes have expression profiles that closely resemble *CFAP70* and *SPEF2* as shown in panel B.

bioRxiv preprint doi: https://doi.org/10.1101/2025.11.10.687652; this version posted November 12, 2025. The copyright holder for this preprint (which was not certified by peer review) is the author/funder, who has granted bioRxiv a license to display the preprint in perpetuity. It is made available under aCC-BY 4.0 International license.

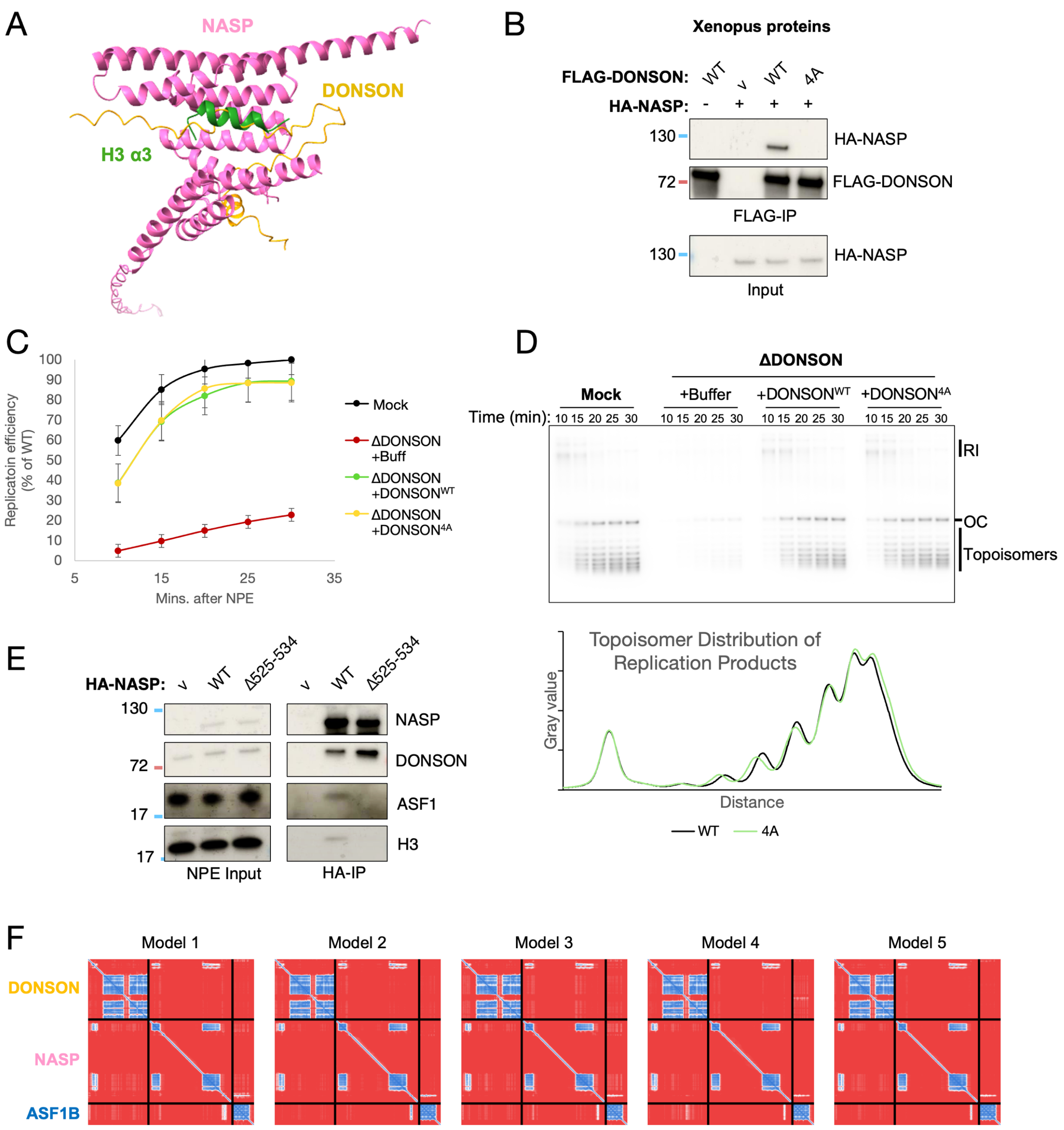

Figure S7: Mechanistic insights into DONSON's interaction with NASP

A. Superimposition of the AF-M predicted DONSON–NASP complex with the NASP–H3 crystal structure (PDB: 7V1L). The overlay illustrates that the DONSON-binding surface on NASP overlaps with the H3-H4-binding interface, indicating that these interactions are mutually exclusive. **B.** Co-immunoprecipitation of *Xenopus* NASP with the indicated FLAG-DONSON variants. Same experiment as in Figure 6D, except using *Xenopus* NASP. **C.** The interaction of DONSON with NASP is not required for efficient DNA replication. DONSON was immunodepleted from egg extracts (NPE), and recombinant CDK2–Cyclin E1 (to restore efficient replication due to partial CDK2–Cyclin E co-depletion) and buffer or the indicated mutants were added back. Licensing and replication initiation were carried out in the presence of radioactive dATP, and replication efficiency was measured by running replication products on native agarose gels and performing autoradiography (see Methods). Data represent mean ± SD (n = 3). **D.** Same experiment as in (C), but plasmids were separated on native gels containing 1 μM chloroquine to resolve plasmid topoisomers, and the autoradiograph of the gel is shown (top), together with a density trace through each lane (bottom), to indicate that there is no difference in the degree of plasmid supercoiling in the different conditions where replication was observed, consistent with there being no major defect in replication-coupled chromatin assembly. **E.** Independent replicate of the data shown in Figure 6F. **F.** Predicted aligned error (PAE) plots of the AF-M predicted DONSON–NASP–ASF1B complex. The color scale indicates the per-residue predicted error, with blue representing low error and red representing high error.

bioRxiv preprint doi: https://doi.org/10.1101/2025.11.10.687652; this version posted November 12, 2025. The copyright holder for this preprint (which was not certified by peer review) is the author/funder, who has granted bioRxiv a license to display the preprint in perpetuity. It is made available under aCC-BY 4.0 International license.

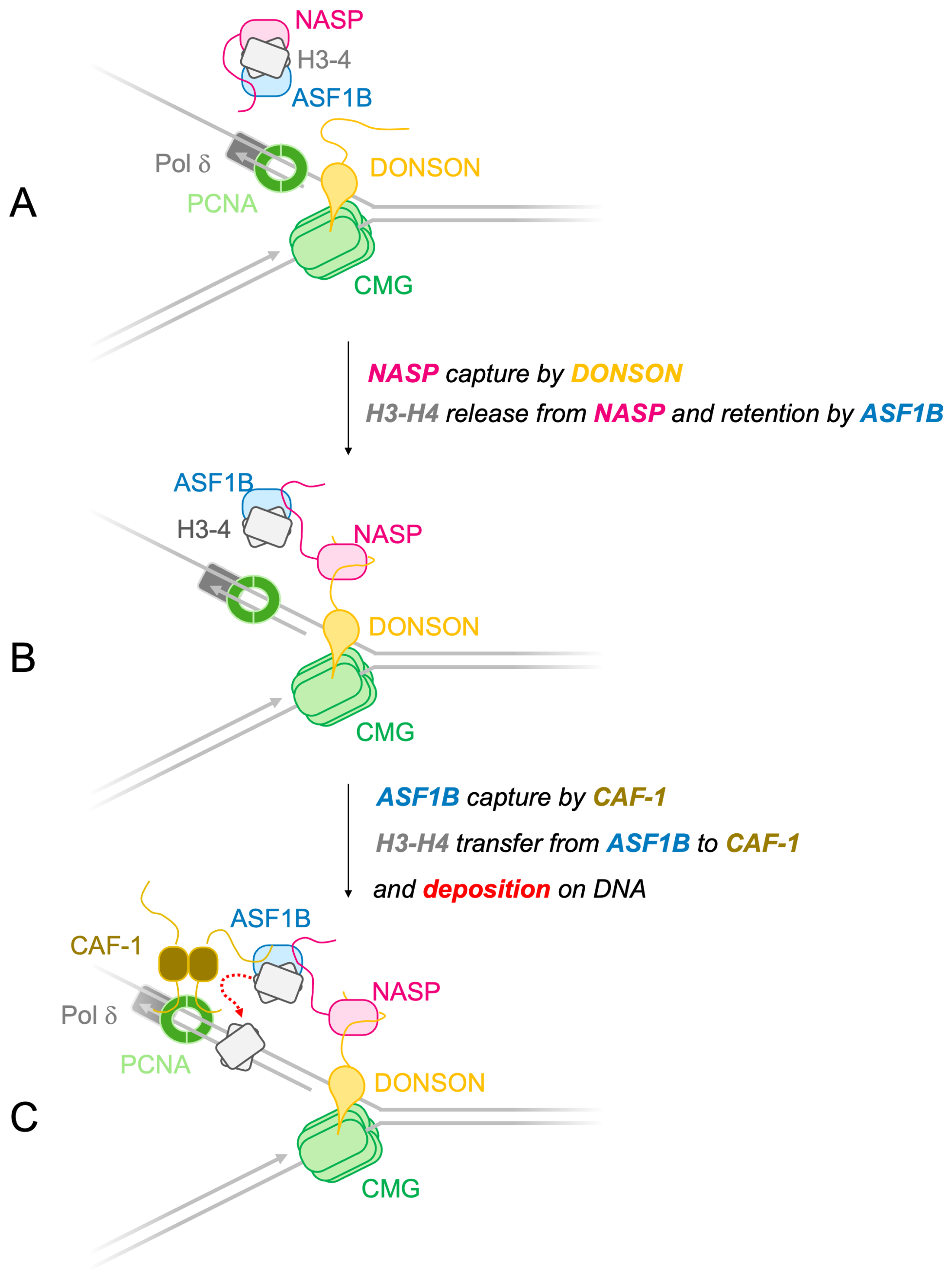

Figure S8: Hypothetical model of DONSON's role in chromatin assembly
Given the new protein-protein interactions reported here (DONSON–NASP and NASP–ASF1B) and the similar effects of DONSON, NASP, and CAF-1 depletion on fork progression, we hypothesize that these proteins cooperate in replication-coupled chromatin assembly, according to the following mechanism. **A.** Our data indicate that NASP and ASF1B bind cooperatively to H3-H4 because a NASP mutant that disrupts the newly identified NASP–ASF1B interaction also no longer binds histone H3. We further propose that DONSON interacts with the replisome through previously described interactions with GINS and MCM3. **B.** We propose that DONSON's N-terminal region captures NASP. Because DONSON and histones bind the same surface on NASP, DONSON binding triggers histone transfer from NASP to ASF1B. Consistent with this model, NASP mutants that lose the interaction with histones exhibit a modest but reproducible increase in binding to DONSON (**Figures 6F and S7E**). The new interaction detected between NASP and ASF1B indicates that the ASF1B–H3-H4 complex might remain associated with NASP after DONSON binding. **C.** The known interaction between CAF-1 and ASF1B facilitates histones transfer from ASF1B to CAF-1, followed by deposition on DNA. We speculate that replication-coupled chromatin assembly remains intact in egg extracts when the DONSON–NASP interaction is disrupted because the large pool of free histones in this system bypasses the need for this pathway. Alternatively, direct interaction with CAF-1 or DONSON–NASP might represent redundant mechanisms for ASF1B–H3-H4 complexes to be concentrated near CAF-1.
